# Identification of *lptA, lpxE*, and *lpxO*, Three Genes Involved in the Remodeling of *Brucella* Cell Envelope

**DOI:** 10.3389/fmicb.2017.02657

**Published:** 2018-01-10

**Authors:** Raquel Conde-Álvarez, Leyre Palacios-Chaves, Yolanda Gil-Ramírez, Miriam Salvador-Bescós, Marina Bárcena-Varela, Beatriz Aragón-Aranda, Estrella Martínez-Gómez, Amaia Zúñiga-Ripa, María J. de Miguel, Toby Leigh Bartholomew, Sean Hanniffy, María-Jesús Grilló, Miguel Ángel Vences-Guzmán, José A. Bengoechea, Vilma Arce-Gorvel, Jean-Pierre Gorvel, Ignacio Moriyón, Maite Iriarte

**Affiliations:** ^1^Universidad de Navarra, Facultad de Medicina, Departamento de Microbiología y Parasitología, Instituto de Salud Tropical (ISTUN) e Instituto de Investigación Sanitaria de Navarra (IdISNA), Pamplona, Spain; ^2^Instituto de Agrobiotecnología, Consejo Superior de Investigaciones Científicas – Universidad Pública de Navarra – Gobierno de Navarra, Pamplona, Spain; ^3^Unidad de Producción y Sanidad Animal, Instituto Agroalimentario de Aragón, Centro de Investigación y Tecnología Agroalimentaria de Aragón – Universidad de Zaragoza, Zaragoza, Spain; ^4^Wellcome-Wolfson Institute for Experimental Medicine, Queen’s University Belfast, Belfast, United Kingdom; ^5^Institut National de la Santé et de la Recherche Médicale, U1104, Centre National de la Recherche Scientifique UMR7280, Centre d’Immunologie de Marseille-Luminy, Aix-Marseille University UM2, Marseille, France; ^6^Centro de Ciencias Genómicas, Universidad Nacional Autónoma de México, Cuernavaca, Mexico

**Keywords:** lipopolysaccharide, *Brucella*, lipids, cell envelope, PAMP

## Abstract

The brucellae are facultative intracellular bacteria that cause a worldwide extended zoonosis. One of the pathogenicity mechanisms of these bacteria is their ability to avoid rapid recognition by innate immunity because of a reduction of the pathogen-associated molecular pattern (PAMP) of the lipopolysaccharide (LPS), free-lipids, and other envelope molecules. We investigated the *Brucella* homologs of *lptA, lpxE*, and *lpxO*, three genes that in some pathogens encode enzymes that mask the LPS PAMP by upsetting the core-lipid A charge/hydrophobic balance. *Brucella lptA*, which encodes a putative ethanolamine transferase, carries a frame-shift in *B. abortus* but not in other *Brucella* spp. and phylogenetic neighbors like the opportunistic pathogen *Ochrobactrum anthropi.* Consistent with the genomic evidence, a *B. melitensis lptA* mutant lacked lipid A-linked ethanolamine and displayed increased sensitivity to polymyxin B (a surrogate of innate immunity bactericidal peptides), while *B. abortus* carrying *B. melitensis lptA* displayed increased resistance. *Brucella lpxE* encodes a putative phosphatase acting on lipid A or on a free-lipid that is highly conserved in all brucellae and *O. anthropi.* Although we found no evidence of lipid A dephosphorylation, a *B. abortus lpxE* mutant showed increased polymyxin B sensitivity, suggesting the existence of a hitherto unidentified free-lipid involved in bactericidal peptide resistance. Gene *lpxO* putatively encoding an acyl hydroxylase carries a frame-shift in all brucellae except *B. microti* and is intact in *O. anthropi*. Free-lipid analysis revealed that *lpxO* corresponded to *olsC*, the gene coding for the ornithine lipid (OL) acyl hydroxylase active in *O. anthropi* and *B. microti*, while *B. abortus* carrying the *olsC* of *O. anthropi* and *B. microti* synthesized hydroxylated OLs. Interestingly, mutants in *lptA, lpxE*, or *olsC* were not attenuated in dendritic cells or mice. This lack of an obvious effect on virulence together with the presence of the intact homolog genes in *O. anthropi* and *B. microti* but not in other brucellae suggests that LptA, LpxE, or OL β-hydroxylase do not significantly alter the PAMP properties of *Brucella* LPS and free-lipids and are therefore not positively selected during the adaptation to intracellular life.

## Introduction

Brucellosis is the collective name of a group of zoonotic diseases afflicting a wide range of domestic and wild mammals (Whatmore, 2009; Zheludkov and Tsirelson, 2010). In domestic livestock brucellosis is manifested mostly as abortions and infertility, and contact with infected animals and consumption of unpasteurized dairy products are the sources of human brucellosis, an incapacitating condition that requires prolonged antibiotic treatment (Zinsstag et al., 2011). Eradicated in a handful of countries, brucellosis is endemic or even increasing in many areas of the world (Jones et al., 2013; Ducrotoy et al., 2017; Lai et al., 2017).

**FIGURE 1:**
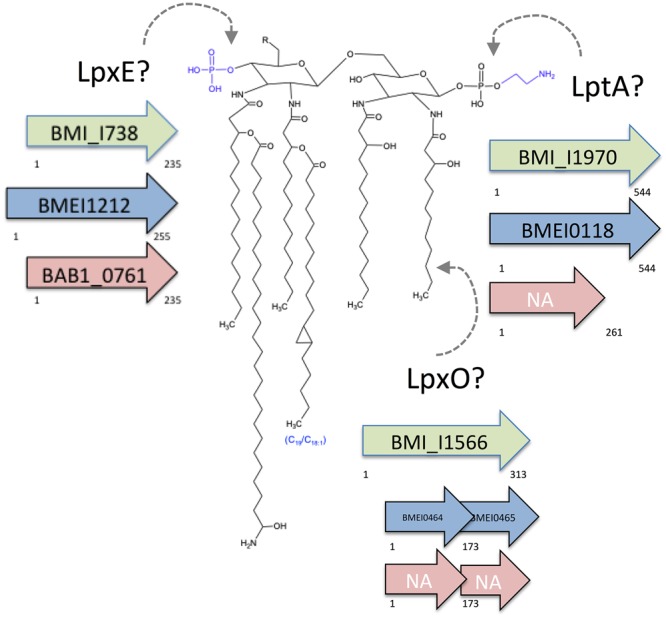
*Brucella* lipid A and hypothetical sites of action of putative LpxE, LptA, and LpxO. The structure proposed is based on acyl-chain and mass spectrophotometry analyses and genomic predictions. The predicted sites of action of LpxE (phosphatase), LptA (pEtN transferase), and LpxO (acyl chain hydroxylase) are indicated, and the corresponding ORF of *B. microti* (green), *B. melitensis* (blue), and *B. abortus* (red) presented (NA, not annotated). The *B. abortus lptA* homolog and the *B. melitensis* and *B. abortus lpxO* homologs carry a frame-shift mutation.

This disease is caused by facultative intracellular parasites of the genus *Brucella*. Taxonomically placed in the α-2 *Proteobacteria* (Moreno et al., 1990), the brucellae are close to plant pathogens and endosymbionts such as *Agrobacterium, Sinorhizobium*, and *Rhizobium* and to soil bacteria such as *Ochrobactrum*, the latter including some opportunistic pathogens, and comparative analyses suggest that soil bacteria of this group are endowed with properties that represent a first scaffold on which an intracellular life style develops (Velasco et al., 2000; Moreno and Moriyón, 2007; Barquero-Calvo et al., 2009). The brucellae owe their pathogenicity mainly to their ability to multiply within dendritic cells, macrophages, and a variety of other cells. Due to their ability to control intracellular trafficking and be barely detected by innate immunity, these bacteria are able to reach a safe intracellular niche before an effective immune response is mounted, and to multiply extensively (Gorvel and Moreno, 2002; Barquero-Calvo et al., 2007). A mechanism used by *Brucella* to scape from the host immune response is the interference with the toll-like receptor (TLR) signaling pathway by the injection of active effectors such as BtpA and BtpB through the Type IV secretion system T4SS. Both effector proteins contain a TIR domain that interferes with TLR signaling by directly interacting with MyD88 (Cirl et al., 2008; Salcedo et al., 2008, 2013; Chaudhary et al., 2012) and contribute to the control of dendritic cell (DC) activation during infection. Moreover, *Brucella* has modified outer membrane (OM) components in order to reduce the pathogen-associated molecular patterns (PAMP) of the cell envelope. In Gram-negative bacteria, these PAMP are created by the conserved composition of the OM lipopolysaccharide (LPS) and the free lipids on which the topology of the OM also depends. However, in addition to free-lipid species present in most Gram-negative bacteria (i.e., cardiolipin, phosphatidylglycerol, and phosphatidylethanolamine), *Brucella* also possesses phosphatidylcholine and amino lipids. Phosphatidylcholine is a eukaryotic-type phospholipid required for *Brucella* full virulence (Comerci et al., 2006; Conde-Alvarez et al., 2006). Among the amino lipids, only the ornithine lipids (OL) have been investigated which unlike their counterparts in *Bordetella*, do not trigger the release of IL-6 or TNF-α by macrophages, possibly on account of their longer acyl chains that reduce the OL PAMP (Palacios-Chaves et al., 2011). Concerning the LPS, most bacteria carry C1 and C4′ glucosamine disaccharides with C12 and C14 acyl and acyl-oxyacyl chains. This highly amphipathic structure, named lipid A, is adjacent to additional negatively charged groups of the core oligosaccharide, namely the heptose phosphates and 2-keto-3-deoxyoctulosonate carboxyl groups (Kastowsky et al., 1992; Moriyón, 2003). This lipid A-core PAMP is so efficiently detected by the innate immunity system that some pathogens partially conceal it by removing phosphate groups or substituting them with arabinosamine and/or ethanolamine, or by hydroxylating the acyl chains (Takahashi et al., 2008; Lewis et al., 2013; Moreira et al., 2013; Needham and Trent, 2013; Llobet et al., 2015; Trombley et al., 2015). In contrast, *Brucella* lipid A is a diaminoglucose disaccharide amide-linked to long (C16, C18) and very long (C28–C30) acyl chains (Velasco et al., 2000; Iriarte et al., 2004; Fontana et al., 2016). Furthermore, negative charges in lipid A phosphates and 2-keto-3-deoxyoctulosonate are counterbalanced by four glucosamine units present in the core (Kubler-Kielb and Vinogradov, 2013; Fontana et al., 2016). As illustrated by the unusually reduced endotoxicity of the *Brucella* LPS this structure is defectively detected by the innate immune response (Lapaque et al., 2005; Martirosyan et al., 2011; Conde-Álvarez et al., 2012). It remains unknown, however, whether *Brucella* LPS undergoes post-synthetic modifications that have been described for other bacteria that could alter its PAMP potential and contribution to virulence. In this work, we investigated in *Brucella* the role of gene homologs to phosphatases, phospho-ethanolamine (pEtN) transferases, and acyl hydroxylases (**Figure 1**) that have been shown in other Gram-negative pathogens to act on LPS and to contribute to overcoming innate immunity defenses.

## Materials and Methods

### Bacterial Strains and Growth Conditions

The bacterial strains and plasmids used in this study are listed in Supplementary Table S1. Bacteria were routinely grown in standard tryptic soy broth or agar either plain or supplemented with kanamycin at 50 μg/ml, or/and nalidixic at 5 or 25 μg/ml or/and 5% sucrose. All strains were stored in skim milk at -80°C.

### DNA Manipulations

Genomic sequences were obtained from the Kyoto Encyclopedia of Genes and Genomes (KEGG) database^1^. Searches for DNA and protein homologies were carried out using the National Center for Biotechnology Information (NCBI^2^) and the European Molecular Biology Laboratory (EMBL) – European Bioinformatics Institute server^3^. Primers were synthesized by Sigma-Genosys (Haverhill, United Kingdom). DNA sequencing was performed by the “Servicio de Secuenciación del Centro de Investigación Médica Aplicada” (Pamplona, Spain). Restriction–modification enzymes were used under the conditions recommended by the manufacturer. Plasmid and chromosomal DNA were extracted with Qiaprep Spin Miniprep (Qiagen) and Ultraclean Microbial DNA Isolation Kits (Mo Bio Laboratories), respectively. When needed, DNA was purified from agarose gels using the Qiack Gel Extraction Kit (Qiagen).

### Mutagenesis

To obtain *BmeΔlptA, BaΔlpxE*, and *BmiΔolsC* in-frame deletion mutants, directed mutagenesis by overlapping PCR were performed using genomic DNA as template and pJQK (Scupham and Triplett, 1997) as the suicide vector. The corresponding gene was deleted using allelic exchange by double recombination as previously described (Conde-Alvarez et al., 2006).

For the construction of the *BmeΔlptA* mutant, we first generated two PCR fragments: oligonucleotides *lptA-*F1 (5′-GAACGCGAGACTATGGAAAC-3′) and *lptA-*R2 (5′-TGGTGAACGCCAGAAGATAGA-3′) were used to amplify a 400-bp fragment including codons 1–26 of *BmelptA* ORF, as well as 324 bp upstream of the *BmelptA* start codon, and oligonucleotides *lptA-*F3 (5′-TCTATCTTCTGGCGTTCACCGCACGACAATCTCTTC-3′) and *lptA*-R4 (5′-AATATTCCATGGCGCATTTC-3′) were used to amplify a 472-bp fragment including codons 506–544 of the *lptA* ORF and 353-bp downstream of the *lptA* stop codon. Both fragments were ligated by overlapping PCR using oligonucleotides *lptA*-F1 and *lptA*-R4 for amplification, and the complementary regions between *lptA*-R2 and *lptA*-F3 for overlapping. The resulting fragment, containing the *lptA* deleted allele, was cloned into pCR2.1 (Invitrogen, Barcelona, Spain), sequenced to ensure maintenance of the reading frame, and subcloned into the *Bam*HI and the *Xba*I sites of the suicide plasmid pJQK. The resulting mutator plasmid (pRCI-32) was introduced in *B. melitensis 16M* by conjugation using the *Escherchia coli* S.17 strain (Simon et al., 1983).

For the construction of the *BaΔlpxE* mutant, we first generated two PCR fragments: oligonucleotides *lpxE*-F1 (5′-CGCGTGTGCCATAGGTATATT-3′) and *lpxE-*R2 (5′-TATAGGCAGGGCGCAGAA-3′) were used to amplify a 482-bp fragment including codons 1–29 of *lpxE* ORF, as well as 394 bp upstream of the *lpxE-*1 start codon, and oligonucleotides *lpxE-*F3 (5′-TTCTGCGCCCTGCCTATAGATTCGTTTCCGCATGGT-3′) and *lpxE-*R4 (5′-CCAATACAC CCGTCATGAGA-3′) were used to amplify a 577-bp fragment including codons 226–255 of the *lpxE* ORF and 488-bp downstream of the *lpxE* stop codon. Both fragments were ligated by overlapping PCR using oligonucleotides *lpxE-*F1 and *lpxE-*R4 for amplification, and the complementary regions between *lpxE*-R2 and *lpxE*-F3 for overlapping. The resulting fragment, containing the *lpxE* deleted allele, was cloned into pCR2.1 (Invitrogen, Barcelona, Spain), sequenced to ensure maintenance of the reading frame, and subcloned into the *Bam*HI and the *Xba*I sites of the suicide plasmid pJQK (Scupham and Triplett, 1997). The resulting mutator plasmid (pRCI-36) was introduced in *B. abortus* 2308 by conjugation using the *E. coli* S.17 strain (Simon et al., 1983).

For the construction of the *BmiΔolsC* mutant, we first generated two PCR fragments: oligonucleotides *olsC-*F1 (5′-TGCTGGATCGTATTCGTCTG-3′) and *olsC-*R2 (5′-GCCATAAGCCGATGGAACTA-3′) were used to amplify a 334-bp fragment including codons 1–15 of *olsC* ORF, as well as 289 bp upstream of the *olsC* start codon, and oligonucleotides *olsC-*F3 (5′-TAGTTCCATCGGCTTATGGCAGGAGGGGCTAGACAACCAC-3′) and *olsC*-R4 (5′-AACCAGCGACAGGGTAAGC-3′) were used to amplify a 320-bp fragment including codons 286–313 of the *olsC* ORF and 237-bp downstream of the *olsC* stop codon. Both fragments were ligated by overlapping PCR using oligonucleotides *olsC*-F1 and *olsC*-R4 for amplification, and the complementary regions between *olsC*-R2 and *olsC*-F3 for overlapping. The resulting fragment, containing the *lptA* deleted allele, was cloned into pCR2.1 (Invitrogen, Barcelona, Spain), sequenced to ensure maintenance of the reading frame, and subcloned into the *Bam*HI and the *Xba*I sites of the suicide plasmid pJQK (Scupham and Triplett, 1997). The resulting mutator plasmid (pRCI-65) was introduced in *B. microti* CM445 by conjugation using the *E. coli* S.17 strain (Simon et al., 1983).

Deletion of each gene was checked with oligonucleotides *gene*-F1 and *gene*-R4 and internal primers hybridizing in the non-deleted regions.

### Complementation of Deleted Genes

For pBME*lpxE* and pBME*lptA* construction we took advantage of the *Brucella* ORFeome constructed with the Gateway cloning Technology (Invitrogen) (Dricot et al., 2004). The clones carrying Bme*lpxE* or Bme*lptA* were extracted and the DNA containing the corresponding ORF was subcloned in plasmid pRH001 (Hallez et al., 2007) to produce pBME*lpxE* and pBME*lptA*. For pBMI*olsC, olsC* was amplified using genomic DNA of Bmi-parental as DNA template. The primers used were *olsC*-F6 (5′-GCTTTCCGAACAAGCACTGA-3′) and *olsC*-R7 (5′-GCCTCCCTTCACCGGTTATT-3′). The resulting PCR product, containing the ORF from 342 bp upstream to 84 bp downstream, was then cloned into pCR2.1 TOPO (Invitrogen) plasmid by “TA cloning” (Life Technologies). The resulting plasmid was sequenced to ensure that the gene was correctly cloned. Then, the gene was subcloned into the *BamHI* and the *XbaI* sites of the replicative plasmid pBBR1 MCS (Kovach et al., 1994) pBME*lpxE*, pBME*lptA*, and pBMI*olsC* were introduced into *Brucella* by conjugation using *E. coli* S.17-1 strain and the conjugants harboring corresponding plasmid were selected by plating onto TSA-Nal-Cm plates.

### Sensitivity to Cationic Peptides

Exponentially growing bacteria were adjusted to an optical density equivalent to one of the McFarland scale and the minimal inhibitory concentrations (MICs) of polymyxin B were determined by the *e*-test method on Müller–Hinton agar (Izasa) or by the serial dilution method in a similar broth.

### LPS Preparation

Lipopolysaccharide was obtained by methanol precipitation of the phenol phase of a phenol–water extract (Leong et al., 1970). This fraction [10 mg/ml in 175 mM NaCl, 0.05% NaN_3_, 0.1 M Tris–HCl (pH 7.0)] was then purified by digestion with nucleases [50 μg/ml each of DNase-II type V and RNase-A (Sigma, St. Louis, MO, United States), 30 min at 37°C] and three times with proteinase K (50 μg/ml, 3 h at 55°C), and ultracentrifuged (6 h, 100,000 × *g*) (Aragón et al., 1996). Free lipids (OLs and phospholipids) were then removed by a fourfold extraction with chloroform–methanol [2:1 (vol/vol)] (Velasco et al., 2000).

### Infections in Mice

Seven-week-old female BALB/c mice (Charles River, Elbeuf, France) were kept in cages with water and food ad libitum and accommodated under biosafety containment conditions 2 weeks before the start of the experiments. To prepare inocula, tryptic soy agar (TSA) grown bacteria were harvested and suspended in 10 mM phosphate buffered saline (pH 6.85), and 0.1 ml/mouse containing approximately 5 × 10^4^ colony forming units (CFU) for *B. melitensis* or *B. abortus* and 1 × 10^4^ CFU for *B. microti* was administered intraperitoneally. The exact doses assessed retrospectively by plating dilutions of the inocula. Number of CFU in spleens was determined at diferent time after inoculation. For this, the spleens were aseptically removed and individually weighed and homogenized in 9 volumes of PBS. Serial 10-fold dilutions of each homogenate were performed and each dilution was plated by triplicate. Plates were incubated at 37°C for 5 days. At several points during the infection process, the identity of the spleen isolates was confirmed by PCR. The individual data were normalized by logarithmic transformation, and the mean log CFU/spleen values and the standard deviations (*n* = 5) were calculated.

### Intracellular Multiplication Assays

Bone marrow cells were isolated from femurs of 7–8-week-old C57Bl/6 female and differentiated into dendritic cells [bone-marrow derived dendritic cells (BMDCs)] as described by Inaba et al. (1992). Infections were performed by centrifuging the bacteria onto the differentiated cells (400 x *g* for 10 min at 4°C; bacteria:cells ratio of 30:1 followed by incubation at 37°C for 30 min under a 5% CO_2_ atmosphere). BMDCs were gently washed with medium to remove extracellular bacteria before incubating in medium supplemented with 50 μg/ml gentamicin for 1 h to kill extracellular bacteria. Thereafter, the antibiotic concentration was decreased to 10 μg/ml. To monitor *Brucella* intracellular survival at different time-points post-infection, BMDC were lysed with 0.1% (vol/vol) Triton X-100 in H_2_O and serial dilutions of lysates were plated onto TSA plates to enumerate the CFU.

### Flow Cytometry

To assess activation and maturation, BMDC were analyzed for surface expression of classical maturation markers at 24 h post-treatment with the different *Brucella* strains and derived mutants. Cells were labeled with fluorochrome-conjugated antibodies specific for mouse CD11c:APC-Cy7 (clone N418), IA-IE:PE (MHC class II clone M5/114.15.2) (PE), CD86:FITC (Clone GL-1), CD40:APC (clone 3/23), and CD80:PE-Cy5 (clone 16-10A1), all from BioLegend. Labeled cells were then subjected to multi-color cytometry using a LSR II UV (Becton Dickinson) and the data analyzed using FlowJo Software by first gating on the CD11c^+^ population (100,000 events) prior to quantifying expression of receptors. Cells were stimulated with *E. coli* LPS (055:B5) as a positive control.

### Lipid A Extraction

Five milligrams of LPS was hydrolyzed in 5 ml 1% acetic acid by sonication, heating to 100°C for 30 min, and cooling to room temperature. Concentrated HCl was added to the mixture until the pH was 1–2. The solution was converted to a two-phase acidic Bligh–Dyer mixture by adding 5.6 ml of chloroform and 5.6 ml of methanol. Phases were mixed by inverting the tubes and separated by centrifugation at 4000 × *g* for 20 min. The lower phases containing lipid A were collected, washed two times with water, and dried under a stream of nitrogen. Extraction was repeated, and the lower phases (11.2 ml) were combined and neutralized with a drop of pyridine. Samples were evaporated to dryness under a stream of nitrogen.

### Mass Spectrometry

Mass spectrometra were acquired on a Bruker Autoflex^®^Speed TOF/TOF Mass Spectrometer (Bruker Daltonics Inc.) in negative reflective mode with delayed extraction. The ion-accelerating voltage was set at 20 kV. Each spectrum was an average of 300 shots. A peptide calibration standard (Bruker Daltonics Inc.) was used to calibrate the Matrix Assisted Laser Desorption/Ionization Time-of-Flight (MALDI-TOF), and lipid A extracted from *E. coli* strain MG1655 grown in LB medium at 37°C.

### Extraction and Analysis of Envelope Lipids

The free-lipid fraction was extracted as described by Bligh and Dyer (1959), and analyzed on a silica gel 60 high-performance thin layer chromatography (HPLC) plates (Merck, Darmstadt, Germany). Chromatography was performed either monodimensionally with chloroform–methanol–water [14:6:1 (volume)] or bidimensionally with chloroform–methanol–water [14:6:1 (volume)] first and chloroform–methanol–acetic acid [13:5:2 (volume)] in the second dimension (Weissenmayer et al., 2002). Plates were developed with 0.2% ninhydrin in acetone at 180°C or 15% sulfuric acid in ethanol at 180°C.

## Results

### The *Brucella lptA* Orthologs Encode a Lipid A Phosphate-Ethanolamine Transferase

A genomic search in the KEGG database revealed that all *Brucella* spp. carry an ORF (BMEI0118 in *B. melitensis*) homologous to *Neisseria meningitidis lptA*, a pEtN transferase that modifies lipid A (Cox et al., 2003). Strikingly, in *B. abortus* but not in other *Brucella* spp., all genomic sequences available at KEGG show a deletion of a thymine in position 774 that should result in a truncated protein lacking the amino acids related to the enzymatic activity (Naessan et al., 2008; **Figure 1** and Supplementary Figure S1 and Supplementary Table S2). In addition to LptA, two other pEtN transferases have been identified in *N. meningitidis*: Lpt-3 and Lpt-6, which, respectively, modify the LPS core at the third and sixth position of heptose II (Mackinnon et al., 2002; Wright et al., 2004). By multiple sequence alignment, the *B. melitensis* putative pEtN transferase showed highest homology with *Neisseria* LptA and also displayed the LptA membrane-associated domains not present in Lpt-3 and Lpt-6 (ORFs NMB1638, NMB2010, and NMA0408, respectively). Accordingly, it can be predicted that ORF BMEI0118 (henceforth BME*lptA*) encodes a pEtN transferase that acts on lipid A, a hypothesis fully consistent with the absence of heptose in the *Brucella* LPS core (Iriarte et al., 2004; Fontana et al., 2016).

**FIGURE 2:**
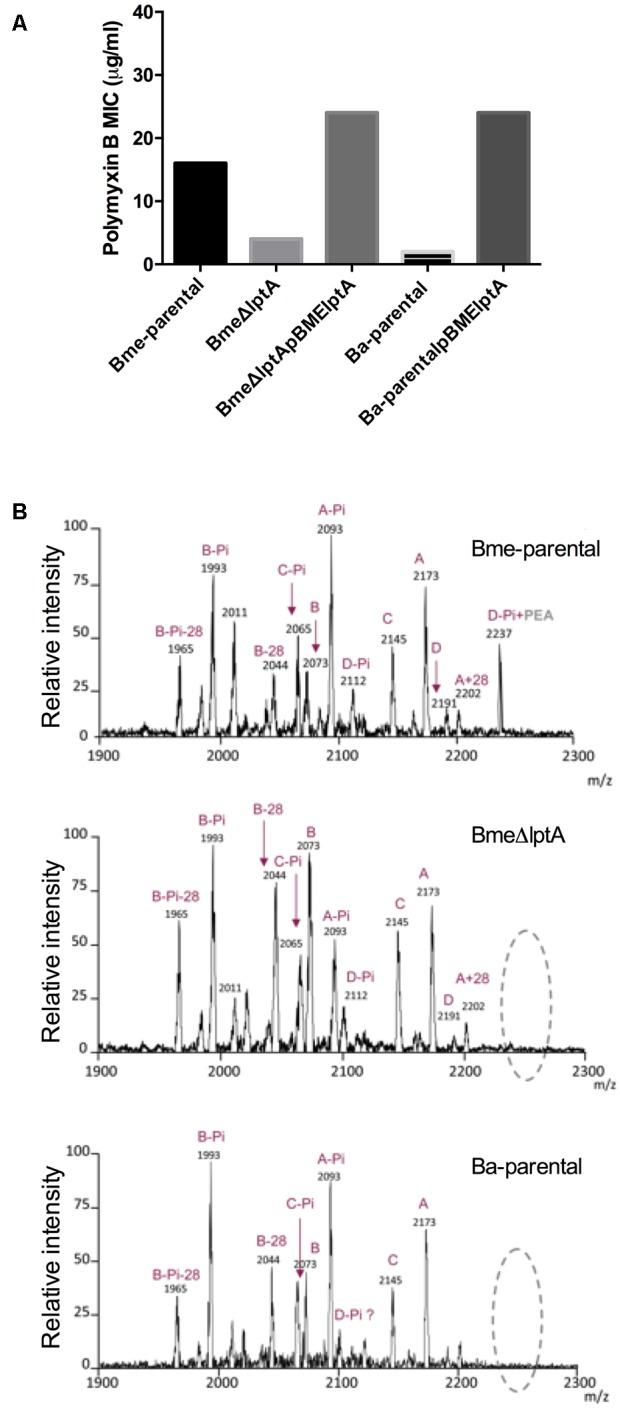
The *Brucella lptA* orthologs are involved in polymyxin B resistance and code for a phosphate-ethanolamine transferase acting on lipid A. **(A)** Polymyxin B sensitivity of *B. melitensis* wild-type (Bme-parental), *B. melitensis* non-polar *lptA* mutant (Bme*lptA*), the cognate complemented mutant (BmeΔ*lptA*pBME*lptA*), *B. abortus* wild-type (Ba-parental), and *B. abortus* wild-type carrying a plasmid with the *B. melitensis lptA* gene (Ba-parentalpBME*lptA*) (the results are representative of three independent experiments). **(B)** MALDI-TOF analysis of the lipid A of Bme-parental, BmeΔ*lptA*, and Ba-parental.

To test this hypothesis, we constructed a *B. melitensis* non-polar mutant (BmeΔ*lptA*) lacking the LptA enzymatic domain (amino acids 26–506), which as expected maintained a smooth (S) phenotype (negative crystal violet test and positive coagglutination with anti-S-LPS antibodies). As a consequence of the increased positive charge of the amino group, pEtN has been shown to decrease binding of the polycationic lipopeptide polymyxin B to LPS, and to increase resistance to this antibiotic in a variety of bacteria (Needham and Trent, 2013; Trombley et al., 2015; Herrera et al., 2017). In keeping with this possibility, the BmeΔ*lptA* mutant was more sensitive to polymyxin B than the parental strain *B. melitensis* 16M (Bme-parental) (**Figure 2A**). In contrast, and consistent with the frame-shift in its *lptA* homolog, *B. abortus* 2308 (Ba-parental) displayed polymyxin B sensitivity similar to that of BmeΔ*lptA*. Moreover, complementation of BmeΔ*lptA* with the multi-copy plasmid pBME*lptA* or its introduction into *B. abortus* 2308 leads to restoration of polymyxin B resistance in BmeΔ*lptA* or an increase up to *B. melitensis* level in *B. abortus* (**Figure 2A**). As expected both constructs kept the S type features (negative crystal violet test and positive coagglutination with anti-S-LPS antibodies) of the parental strains. *N. gonorrhoeae* shows increased resistance to the action of complement in non-immune serum that is dependent on lipid A-linked pEtN (Lewis et al., 2013). Testing for a similar contribution here, we found that BmeΔ*lptA* was more sensitive than either the parental strain or the complemented mutant (25% vs. no decrease in viability after 3 h of incubation in normal sheep serum) relevant given that *B. melitensis* is characteristically resistant to killing by normal serum.

By MALDI-TOF analysis, the lipid A of Bme-parental was found to contain four main clusters of ions (A, B, C, and D in **Figure 2B**). BmeΔ*lptA* lipid A was qualitatively identical to Bme-parental with respect to groups A, B, and C but clearly differed in group D (**Figure 2B** and Supplementary Table S3). In group D, the 2191 m/z″ species of Ba-parental was consistent with the isotopic mass of a molecule (C_120_H_232_N_4_O_25_P_2_) formed by a hexaacylated and bisphosphorylated diaminoglucose disaccharide carrying the hydroxylated long and very long chain acyl groups characteristic of *Brucella* (Velasco et al., 2000; Ferguson et al., 2004). According to this interpretation, the signal(s) at 2112 m/z (mass of - H_2_PO_3_ o - HPO_3_ -, 80.9 - 79.9) could correspond to a monophosphorylated (C_120_H_232_N_4_O_25_P) 2191 m/z″ equivalent. Substitution of this monophosphorylated form with pEtN (^+^H_3_NCH_2_-CH_2_- HPO_3_ mass 125) should account for signal m/z 2237, in keeping with the fact that m/z 2237 did not appear in the spectrum of the lipids A from either BmeΔ*lptA* or Ba-parental (**Figure 2B**). Although a clear cut demonstration requires direct analyses of the enzymatic analyses of LptA, these results and the homologies with LptA of other bacteria are consistent with the hypothesis that LptA acts as a pEtN transferase in *B. melitensis* and lacks functionality in *B. abortus*. It is remarkable that pEtN activity was detected for only a fraction (D) of lipid A species. This could be explained by a preferential activity of the enzyme for higher MW lipid A molecules.

### The *Brucella lpxE* Orthologs Encode a Phosphatase Involved in the Remodeling of the OM

As described above, MALDI-TOF analyses showed the presence of molecular species with a mass compatible with monophosphorylated lipid A. Since lipid A synthesis produces C1 and C4′ bisphosphorylated disaccharide backbones (Qureshi et al., 1994), a possible explanation could be its dephosphorylation by a phosphatase such as LpxE, an inner membrane enzyme that in the phylogenetic neighbor *Rhizobium leguminosarum* removes the lipid A phosphate at C1 (Raetz et al., 2009). A search in KEGG showed that all *Brucella* spp. carry an ORF homologous to *R. leguminosarum lpxE* (Supplementary Table S2). However, the start codon in the *B. melitensis* 16M homolog (BMEI1212) is annotated to a position different from that determined for other brucellae (Supplementary Table S2), including other *B. melitensis* strains. Thus, whereas the *B. abortus* homolog (BAB1_0671) is predicted to encode a protein of 255 amino acids, the *B. melitensis* one could encode a protein of either 235 or 255 amino acids (**Figure 1**). Both proteins conserve the consensus sequence of the lipid phosphatase superfamily [KX6RP-(X12–54)-PSGH-(X31–54)-SRX5HX3D] (Stukey and Carman, 2008) which is also present in LpxE from *R*. *leguminosarum, Sinorhizobium meliloti*, and *Agrobacterium tumefaciens* (Karbarz et al., 2003). Although BAB1_0671 and BMEI1212 code for proteins that contain the three motifs conserved in the LpxF phosphatase from *Francisella*, they lack two amino acids of the central motif, NCSFX2G, which seems LpxF specific (Wang et al., 2006, 2007). Thus, the *Brucella* proteins were named BALpxE and BMELpxE.

To study whether BALpxE actually acts as a lipid A phosphatase, we constructed a non-polar mutant (BaΔ*lpxE*) and tested it against polymyxin B, since the permanence of a phosphate group in an OM molecule should increase sensitivity to this antibiotic. Mutant BaΔ*lpxE* was eight times more sensitive than the parental strain (MIC 0.2 and 1.6 μg/ml, respectively). Moreover, when we introduced a plasmid containing the BME*lpxE* ortholog into BaΔ*lpxE*, the resistance to polymyxin B was restored (MIC 1.6 μg/ml). Although final confirmation of this interpretation would require to assay the enzymatic activity of the protein, these results are consistent with the predicted role of *lpxE* as a phosphatase and its functionality in both *B. abortus* and *B. melitensis* 16M, a strain where the annotation of the start codon was a source of ambiguity.

By MALDI-TOF analysis, the Ba-parental lipid A spectrum showed three of the four predominant clusters of ions (A, B, and C) found in *B. melitensis* (**Figure 2B** and Supplementary Table S3). Cluster A (m/z 2173) was consistent with an hexaacylated bisphoshorylated diaminoglucose disaccharide (C_120_H_232_N_4_O_24_P_2_) and the signal at 2093 m/z, which differed in the mass of one phosphate group (i.e., 80), was consistent with the cognate monophosphorylated lipid A (C_120_H_232_N_4_O_25_P) (A-Pi, **Figure 2**). Other signals differing in a mass of 14 or 28 units should result from the heterogeneity in acyl chain length that is typical of lipid A. The B and C clusters also contained signals differing in 80 mass units that could correspond to bis- and mono-phosphorylated species. The mass spectrum of BaΔ*lpxE* lipid A (not shown) did not differ significantly from that of Ba-parental, and again showed acyl chain heterogeneity in the A, B, C clusters, as well as the -80 m/z signals indicative of mono- and bisphoshorylated lipid A species. As mutation of lpxE is concomitant with an increase in polymyxin B sensitivity, it is tempting to speculate that LpxE directly or indirectly modulates *Brucella* cell envelope by removing an accessible phosphate group from a substrate different from lipid A. Further studies need to be performed to clarify the role of LpxE.

### The *Brucella lpxO* Orthologs Encode an Acyl Hydroxylase Acting on Ornithine Lipids

**FIGURE 3:**
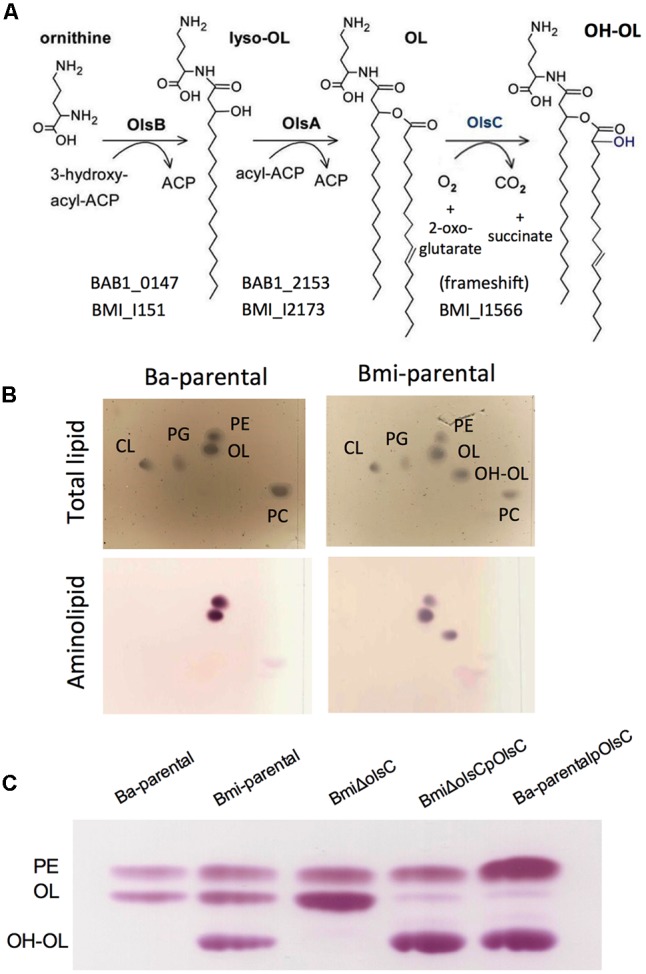
The *Brucella lpxO* orthologs encode an acyl hydroxylase acting on ornithine lipids. **(A)** Pathway of synthesis of ornithine lipids in α-2 *Proteobacteria* (adapted from Geiger et al., 2010); the ORFs of *B. abortus* and *B. microti* are indicated, whereas *B. microti, B. vulpis*, and *O. anthropi* contain an intact *olsC* acyl hydroxylase gene, *B. abortus* and other *Brucella* spp. carry a frame-shift in the *olsC* homolog. **(B)** Lipid profile of *B. abortus* wild-type (Ba-parental) and *B. microti* wild-type (Bmi-parental) showing the absence or presence, respectively, of OH–OL. **(C)** Amino lipid profile of *B. abortus* wild-type (Ba-parental), *B. microti* wild-type (Bmi-parental), *B. microti* deleted in *olsC* (BmiΔ*olsC*), the cognate reconstituted mutant (BmiΔ*olsC*pOlsC), and *B. abortus* wild-type carrying a plasmid with the *B. microti olsC* gene (Ba-parentalpOlsC).

The genomes of all *Brucella* species available at KEGG contain an ORF homologous to *Salmonella lpxO* (Gibbons et al., 2000), which encodes an enzyme hydroxylating the 3′-secondary acyl chain of lipid A. In all *Brucella* spp. except *B. microti* and *B. vulpis* this ORF presents a frame-shift leading to a truncated protein that lacks the consensus of the aspartyl/asparaginyl β-hydroxylases family to which LpxO belongs (**Figure 1** and Supplementary Table S2). These characteristics are consistent with chemical studies that previously failed to observe S2 hydroxylated fatty acids in *B. abortus* lipid A (Velasco et al., 2000). Moreover, a *lpxO* homolog is present in *Ochrobactrum anthropi* where S2 hydroxylated fatty acids were also not observed in the lipid A (Velasco et al., 2000), indicating that a role similar to that of *Salmonella* LpxO is unlikely. Thus, the *lpxO* homologs present in these *B. microti* and *O. anthropi* could be acting on a free lipid and, in fact, it has been reported that the corresponding *R. tropici* homolog is a β-hydroxylase acting on OLs (Vences-Guzmán et al., 2011). If this were the case in *O. anthropi* and the brucellae, the end product [a hydroxylated OL (OH–OL)] of the pathway described previously in members of the *Rhizobiaceae* (**Figure 3A**) should be observed in *O. anthropi* and *B. microti* (and *B. vulpis*) but not in other *Brucella* spp.

To investigate these hypotheses, we compared the free lipids of *B. abortus, B. melitensis, B. suis, B. ovis, B. microti*, and *O. anthropi*. As can be seen in **Figure 3B**, *B. microti* but not *B. abortus* produced an amino lipid with the migration pattern predicted for OH-OL (Vences-Guzmán et al., 2011), and results similar to those of *B. microti* were obtained for *O. anthropi* but not for the other *Brucella* spp. tested (not shown). These observations support the interpretation that *O. anthropi* and *B. microti* LpxO are OL hydroxylases and are fully consistent with the aforementioned genomic and chemical evidence. Accordingly, *Brucella lpxO* should be named *olsC.* To confirm this, we examined the amino lipids of a non-polar *olsC* mutant in *B. microti* (BmiΔ*olsC*). As predicted, this mutant did not synthesize OH–OL and complementation with a plasmid containing *B. microti olsC* restored the wild-type phenotype (**Figure 3C**). Furthermore, introducing this plasmid or a plasmid carrying *O. anthropi olsC* into *B. abortus* resulted in the synthesis of OH–OL (**Figure 3C** and Supplementary Figure S2). No difference in polymyxin sensitivity was observed in these constructs or the mutant BmiΔolsC when compared to the corresponding parental strains.

### LptA, LpxE, and OlsC Are Not Required for *Brucella* Virulence in Laboratory Models

**FIGURE 4:**
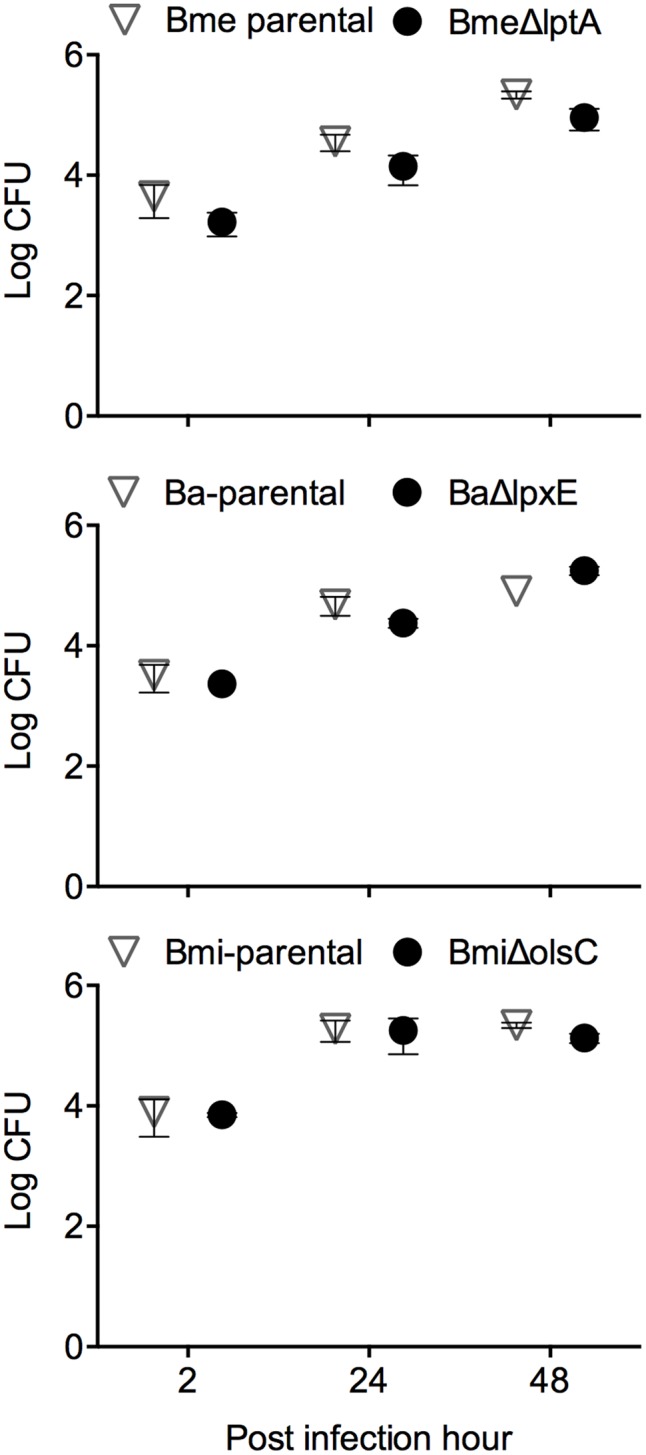
LptA, LpxE, and OlsC deletions do not alter the *Brucella* interaction with dendritic cells. Intracellular replication in BMDCs (each point represents the mean ± standard error of the logarithm of CFU in dendritic cells).

**FIGURE 5:**
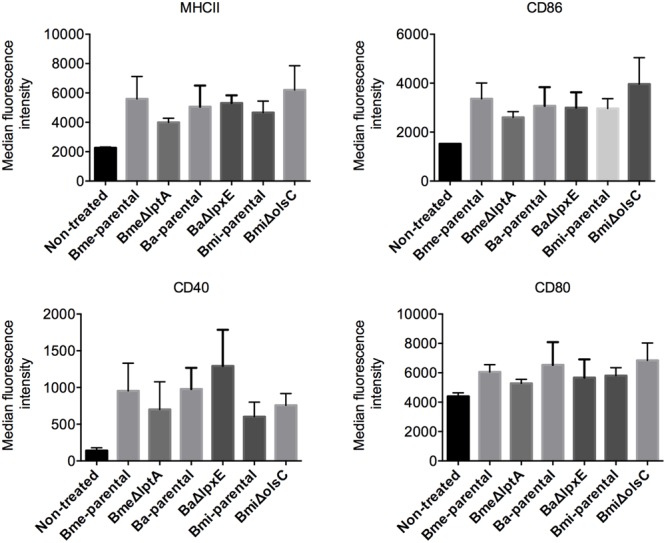
LptA, LpxE, and OlsC deletions do not significantly impact the intrinsic immunogenicity of *Brucella*. Each point represents the mean ± standard error of the median intensity of surface receptor expression in dendritic cells treated with *Brucella* strains or derived mutants. *E. coli* LPS was used as a positive control for dendritic cell activation.

*Brucella abortus, B. melitensis*, and *B. suis* have been shown to multiply in murine and human monocyte-derived dendritic cells while interfering with their activation and maturation and reducing both antigen presentation and an effective adaptive response (Billard et al., 2007; Martirosyan et al., 2011; Conde-Álvarez et al., 2012; Gorvel et al., 2014; Papadopoulos et al., 2016). To assess whether LptA, LpxE, and OL β-hydroxylase (OlsC) were involved, we compared parental and mutant strains of *B. melitensis, B. abortus*, and *B. microti* in mouse BMDCs. As shown in **Figure 4**, the kinetics of multiplication of the mutants and wild-type strains were similar. We also performed a phenotypic characterization of MHC II and co-stimulatory receptors CD86 and CD80 (**Figure 5**). In agreement with previous studies, these analyses showed that activation and maturation was only partially induced in BMDC infected with *B. melitensis* and *B. abortus* (Martirosyan et al., 2011). In addition, a similar partial-activation profile was evident both for *B. microti*, for which no previous studies exist in infected BMDC, and all of the tested mutants obtained for each of the three *Brucella* spp.

**FIGURE 6:**
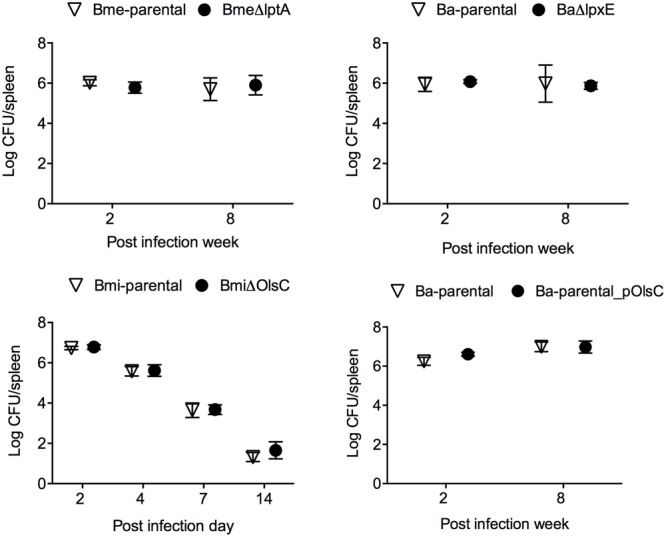
The OM properties that depend on LptA, LpxE, and OlsC are not required for *Brucella* virulence in the mouse model. BALB/c mice were inoculated intraperitoneally with 5 × 10^4^ (Bme-parental, BmeΔlptA, Ba-parental, and BaΔlpxE) or 1 × 10^4^ (Bmi-parental and BmiΔolsC) CFU/mouse and CFU/spleen determined at the indicated times. Each point represents the mean ± standard error of the logarithm of CFU in the spleens of five animals.

The mouse model has been widely used for testing *Brucella* virulence (Grilló et al., 2012). In this model, the LptA and LpxE mutants and the parental strains behaved identically (**Figure 6** upper panels). Deletion of *olsC* in *B. microti* did not alter the CFU/spleen profile produced by this species which is characterized by a lower lethal dose in mice as well as a faster clearance from mouse spleens (Jiménez de Bagüés et al., 2010; **Figure 6**, lower left panel). Moreover, when we tested whether the expression of *B. microti olsC* in *B. abortus* could affect virulence, we found no differences between the *B. microti olsC*-carrying and the wild-type *B. abortus* strains (**Figure 6**, lower right panel).

## Discussion

In this work we investigated three *Brucella* ORFs that according to homologies with genes of known function in other pathogens could modify the lipid A and contribute to further altering the LPS PAMP of representative *Brucella* species. The results show that, whereas *Brucella* LptA modifies the lipid A, this is not the case for *lpxE* and *lpxO* (redesignated *olsC*), the former encoding a putative phosphatase acting on an unidentified OM molecule and the latter for an enzyme with OlsC activity.

Our data strongly suggest that *B. melitensis* LptA is involved in the addition of pEtN to lipid A, homologous proteins carrying out this function are not uncommon in Gram-negative pathogens and modulate the properties of lipid A. In *Salmonella* Typhimurium, *Shigella flexneri, E. coli, Vibrio cholerae, Helicobacter pylori, Haemophilus ducreyi, N. gonorrhoeae*, and *N. meningitidis* pEtN reduces the binding of cationic bactericidal peptides by balancing the negative charge of lipid A (Needham and Trent, 2013; Trombley et al., 2015). Conversely, pEtN promotes binding to *N. gonorrhoeae* lipid A of factors that downregulate the complement cascade and thwart building of the membrane-attack complex and opsonophagocytosis (Lewis et al., 2013). *N. meningitidis* pEtN also promotes adhesion of non-encapsulated bacteria to endothelial cells (Takahashi et al., 2008). Indeed, properties that parallel some of those observed for the above-listed pathogens can also be attributed to the pEtN transferase counterpart in *Brucella*. An intact *lptA* was related to polymyxin B resistance in *B. melitensis* and the introduction of *B. melitensis lptA* into *B. abortus* increases polymyxin B resistance to the level of *B. melitensis*, suggesting that LptA function is severally impaired in *B. abortus*. This is in agreement with the presence of a frame-shift in *B. abortus lptA* encompassing the consensus sequence, which makes likely that it codes for a protein with no or residual enzymatic activity. Previous analyses are contradictory with regard to the presence (Casabuono et al., 2017) or absence (Moreno et al., 1990) of ethanolamine in *B. abortus* lipid A but the materials analyzed differ in methods of extraction and presence of *B. abortus* lipid A markers, such as very long chain fatty acids (VCLFA). Although further chemical and enzymatic analyses are necessary for a definite conclusion, our results strongly suggest that, if present, pEtN is in much less amounts in *B. abortus* than in *B. melitensis* lipid A. It is also worth noting that such genetic and phenotypic differences in the lipid A of *B. abortus* and *B. melitensis* could relate to differences in biological properties. The LPS of *B. abortus* and *B. melitensis* is a poor activator of the complement cascade, and this property has been traced to the core and lipid A structure (Moreno et al., 1981; Conde-Álvarez et al., 2012; Fontana et al., 2016). Since *B. abortus* is less resistant than *B. melitensis* to normal serum (González et al., 2008), it is tempting to suggest that, like in *N. gonorrhoeae, B. melitensis* pEtN could sequester regulatory elements enhancing complement resistance in this species.

Concerning LpxE, phosphatases acting on lipid A have at least been shown in *Francisella tularensis, H. pylori, Porphyromonas gingivalis*, and *Capnocytophaga canimorsus*, bacteria where lipid A dephosphorylation is involved both in resistance to bactericidal peptides and the reduction of TLR-4-dependent recognition (Needham and Trent, 2013). Although these properties are displayed by the LPS of *B. abortus* and *B. melitensis* (Martínez de Tejada et al., 1995; Lapaque et al., 2006; Conde-Álvarez et al., 2012), our results do not support a role for BALpxE as a lipid A phosphatase. This is consistent with genomic analysis showing that, whereas in bacteria where LpxE acts on lipid A the gene is located together with *lptA* in an operon (Tran et al., 2006; Renzi et al., 2015), *Brucella lpxE* is instead located upstream of three sequences annotated as pseudogenes and downstream, but in the opposite direction, of a cystathionine beta-lyase. On the basis of the data shown here, the origin of monophosphoryl lipid A in *Brucella* remains to be explained. Further, we believe it unlikely to be an artifact resulting from the hydrolytic steps used to obtain lipid A and instead favor the hypothesis of the existence of an as yet unidentified lipid A phosphatase.

LpxE belongs to the type 2 family of phosphatases that can act on lipid A but also on phosphatidylglycerol phosphate, phosphatidic acid, sphingosine phosphate, and lysophosphatidic acid (Brindley and Waggoner, 1998; Sciorra and Morris, 2002). Significantly, LpxE from *Agrobacterium*, although predicted to be a lipid A phosphatase, dephosphorylates phosphatidyl glycerophosphate (Karbarz et al., 2009) to generate phosphatidylglycerol, a cell envelope phospholipid. Indeed, a hypothetical phosphatidyl glycerophosphate phosphatase activity of *Brucella* LpxE could account for both the polymyxin B sensitivity of the mutated bacteria and the unaltered mass spectra of the lipid A of the mutant. Such a modification of a phospholipid could be meaningful by itself on account of the LpxE-dependent bactericidal peptide resistance but there are other possibilities. In some bacteria (i.e., *Rhizobium*) phosphatidylglycerol is a precursor for the synthesis of amino lipids such as lysyl-phosphatidylglycerol. This synthesis is induced by acid pH and brings about resistance to daptomycin and polymyxin B (Sohlenkamp et al., 2007; Ernst and Peschel, 2011; Arendt et al., 2012). Interestingly, whereas the BaΔ*lpxE* mutant is impaired for growth at pH 6, the parental *B. abortus* becomes more resistant to cationic peptides (L. Palacios-Chaves and R. Conde-Álvarez, Unpublished observations). These observations suggest the existence in *Brucella* of pH-dependent envelope modifications that require a functional LpxE. Research is in progress to elucidate the mechanisms behind the increased resistance at acid pH and the implication regarding a role for LpxE.

In *S.* Typhimurium, *Pseudomonas aeruginosa, Bordetella bronchispetica, Legionella pneumophila*, and *Klebsiella pneumonia*, LpxO is a Fe2+/α-ketoglutarate-dependent dioxygenase that catalyzes the hydroxylation of the 3′-secondary acyl chain of lipid A. LpxO has been implicated indirectly in stress responses at the envelope level (Needham and Trent, 2013) and, in *K. pneumoniae*, it has been shown to be relevant *in vivo* by increasing bactericidal peptide resistance and reducing the inflammatory responses (Llobet et al., 2015). However, as discussed above, previous chemical analysis (Velasco et al., 2000) of lipid A and the evidence presented here indicate that the *Brucella lpxO* homolog is not a lipid A hydroxylase but rather an OlsC whose mutation, in contrast with LpxO, does not result in increased sensitivity to polymyxin B. This absence of an effect on polycation resistance is in keeping with both the lack of activity on lipid A and the fact that OL do no play a major role in resistance to polycationic bactericidal peptides in *B. abortus* (Palacios-Chaves et al., 2011). At the same time, it would also appear to rule out, the involvement of this protein in the metabolism of succinate in *B. microti* as has been previously suggested (Audic et al., 2009).

Previous data showing *lptA, lpxE*, and *lpxO* to be involved in modulating the properties of the OM in a way that in some cases confers *in vitro* resistance to innate immunity bactericidal peptides, complement, and cytokine responses (Needham and Trent, 2013) have been drawn upon as evidence for a role in virulence. However, to the best of our knowledge, a role *in vivo* has thus far been shown only for *lpxO* from *K. pneumoniae* (Llobet et al., 2015). Moreover, contrasting results have been obtained with mutants both showing bactericidal peptide sensitivity *in vitro* and no phenotype *in vivo* have been reported for at least *H. ducreyi* (Trombley et al., 2015) and may reflect the complexities of the infection processes and/or the inadequacies of the currently available *in vivo* models. Despite their effect on the envelope, our results show that *Brucella lptA, lpxE*, or *olsC* do not play a role in the ability of *Brucella* to replicate in BMDC and do not modulate the activation and maturation profile in these cells. Similarly, the mouse model did not reveal any effect on its ability to colonize and multiply in the spleen. However, further experimental work in the natural hosts and alternative routes of infection might provide evidence on the role in virulence of these genes. The fact that *lptA* and *olsC* are not functional in all *Brucella* spp. must therefore be considered in the context of the models used. While the absence of a functional *lptA* in *B. abortus* suggests that the gene is not essential for the virulence of this species we cannot conclude it to be totally irrelevant. Differences between *B. melitensis* and *B. abortus* related to *lptA* could explain the higher invasiveness of the former species noted by early researchers in studies carried out in guinea pigs, animals that are highly susceptible to brucellosis (Braude, 1951). This possibility together with the presence of intact *lptA* and *olsC* in *Ochrobactrum* and *B. microti* is also compatible with the hypothesis that they represent ancestral characters that are liable to be lost in the absence of a selective pressure during the intracellular life cycle or, in the case of *lptA*, that is no longer present in the ruminant host species (i.e., cattle) to which *B. abortus* is characteristically associated.

## Ethics Statement

Female BALB/c mice (Charles River, France) were kept in cages with water and food *ad libitum* under P3 biosafety conditions in the facilities of “Centro de Investigación Médica Aplicada” (registration code ES31 2010000132) 2 weeks before and during the experiments. The procedures were in accordance with the current European (directive 86/609/EEC) and Spanish (RD 53/2013) legislations, supervised by the Animal Welfare Committee of the University of Navarra, and authorized by the “Gobierno de Navarra” [CEEA045/12 and E36-14 (045-12E1)].

## Author Contributions

IM, MI, J-PG, JB, and RC-Á conceived the study. RC-Á, LP-C, YG-R, MB-V, MS-B, BA-A, EM-G, AZ-R, MdM, TLB, SH, M-JG, MV-G, and VA-G carried out the experimental work. IM, MI, and RC-Á wrote the paper. All authors participated in the presentation and discussion of results.

## Conflict of Interest Statement

The authors declare that the research was conducted in the absence of any commercial or financial relationships that could be construed as a potential conflict of interest.

## Footnotes

1 http://www.genome.jp/kegg/

2 http://www.ncbi.nlm.nih.gov/

3 http://www.ebi.ac.uk/
