# Supplementary material for: Identification of lptA, lpxE, and lpxO, Three Genes Involved in the Remodeling of Brucella Cell Envelope
Source: Front Microbiol. 2018 Jan 10;8:2657. doi: 10.3389/fmicb.2017.02657 (PMC5767591; doi:10.3389/fmicb.2017.02657)
Supplement: Supplementary file 1 [file Table_1.PDF]

Table S1. Bacterial strains and plasmids.

Strains	Relevant characteristics	Reference
Bme-parental	*Brucella melitensis* 16M virulent biovar 1, smooth LPS, sponataneus NalR (25ug/ml)	(González et al., 2008)
Ba-parental	*Brucella abortus* 2308 virulent biovar 1, smooth LPS, sponataneus NalR (25ug/ml)	(Sangari and Agüero, 1991)
Bmi-parental	*Brucella microti* CM445, smooth LPS, NalR (5ug/ml)	(Scholz et al., 2008)
BmeΔlptA	Bme-parental BMEI0118Δ26-506	This work
BmeΔlptA-pBMELptA	BmeΔ*lptA* harboring plasmid p*lptA* encoding BMEI0118	This work
Ba-parental-pBMELptA	Ba-parental harboring plasmid pBMElptA encoding BMEI0118	This work
BaΔlpxE	Ba-parental BAB1_0761Δ29-226	This work
BaΔlpxE-pBMELpxE	Ba*ΔlpxE* harboring plasmid pBMElpxE encoding BMEI1212	This work
BmiΔolsC	Bmi-parental BMI_I1566Δ15-286	This work
BmiΔolsC-pBMIOlsC	BmiΔ*olsC* harboring plasmid pBMIOlsC encoding BMI_I1566	This work
Ba-parental-pBMIolsC	Ba-parental harboring plasmid pBMIOlsC encoding BMI_I1566	This work
Ba-parental-pOcholsC	Ba-parental harboring plasmid pOchOlsC encoding Oant_1613	This work
Top10F´	F´{lacIq, Tn10(TetR)} *mcrA* Δ(*mrr-hsdRMS-mcrBC*) Φ80*lacZ*ΔM15 Δ*lacX*74 *recA*1 *araD*139 Δ(*ara leu*) 7697 *galU galK rpsL* (StrR) endA1 nupG	Invitrogen
E. coli S17λpir	Mating strain with plasmid RP4 inserted into the chromosome	(Simon et al., 1983)

Plasmids		
pCR2.1 TOPO	Cloning verctor, AmpR, KmR	Invitrogen
pJQK	Derivative of pJQ200 KS +, KmR, GmS	(Scupham and Triplett, 1997)
pRH001	Derivative of pMR10 Km^R; Cm^R containing attR sites for LR reaction (Gateway system)	(Hallez et al., 2007)
pBBR1 MCS CmR	Cloning vector, CmR	(Kovach et al., 1994)
pRCI-32	BMEI0118Δ26-506 cloned into *Bam*HI-*Xho*I sites of pJQK	This work
pRCI-36	BAB1_0761*Δ29-226* cloned into *Bam*HI-*Xho*I sites of pJQK	This work
pRCI-61	BMI_I1566Δ15-286 cloned into *Bam*HI-*Xho*I sites of pJQK	This work
pDONR201 BMEI0118	*B. melitensis* chromosomal DNA containing the complete *lptA* gene, generated by PCR and cloned into pDONR201 (Invitrogen)	(Dricot et al., 2004)
pBMElptA	attL1- attL2 fragment of pDONR201- BMEI0118 cloned into the attR1-attR2 sites of pRH001	This work
pDONR201 BMEI1212	*B. melitensis* chromosomal DNA containing the complete *lpxE* gene, generated by PCR and cloned into pDONR201 (Invitrogen)	(Dricot et al., 2004)
pBMElpxE	attL1- attL2 fragment of pDONR201- BMEI1212 cloned into the attR1-attR2 sites of pRH001	This work
pBMIolsC	BMI_I1566 cloned into *Bam*HI-*Xba*I sites of pBBR1 MSC CmR	This work

Bibliography

Dricot, A., Rual, J.-F., Lamesch, P., Bertin, N., Dupuy, D., Hao, T., et al. (2004). Generation of the *Brucella melitensis* ORFeome version 1.1. *Genome Res.* 14, 2201–2206. doi:10.1101/gr.2456204.

González, D., Grilló, M.-J., de Miguel, M.-J., Ali, T., Arce-Gorvel, V., Delrue, R.-M., et al. (2008). Brucellosis vaccines: assessment of *Brucella melitensis* lipopolysaccharide rough mutants defective in core and O-polysaccharide synthesis and export. *PLoS ONE* 3, e2760. doi:10.1371/journal.pone.0002760.

Hallez, R., Letesson, J.-J., Vandenhaute, J., and De Bolle, X. (2007). Gateway-based destination vectors for functional analyses of bacterial ORFeomes: application to the Min system in *Brucella abortus*. *Appl. Environ. Microbiol.* 73, 1375–1379. doi:10.1128/AEM.01873-06.

Kovach, M. E., Phillips, R. W., Elzer, P. H., Roop, R. M., and Peterson, K. M. (1994). pBBR1MCS: a broad-host-range cloning vector. *Biotechniques* 16, 800–802.

Sangari, F. J., and Agüero, J. (1991). Mutagenesis of *Brucella abortus* : comparative efficiency of three transposon delivery systems. *Microb. Pathog.* 11, 443–446.

Scholz, H. C., Hubalek, Z., Sedlacek, I., Vergnaud, G., Tomaso, H., Dahouk, Al, S., et al. (2008). *Brucella microti* sp. nov., isolated from the common vole Microtus arvalis. *International Journal of Systematic and Evolutionary microbiology* 58, 375–382. doi:10.1099/ijs.0.65356-0.

Scupham, A. J., and Triplett, E. W. (1997). Isolation and characterization of the UDP-glucose 4'-epimerase-encoding gene, galE, from *Brucella abortus* 2308. *Gene* 202, 53–59.

Simon, L. D., Randolph, B., Irwin, N., and Binkowski, G. (1983). Stabilization of proteins by a bacteriophage T4 gene cloned in *Escherichia coli*. *Proc. Natl. Acad. Sci. U.S.A.* 80, 2059–2062.
