# Supplementary material for: Identification of lptA, lpxE, and lpxO, Three Genes Involved in the Remodeling of Brucella Cell Envelope
Source: Front Microbiol. 2018 Jan 10;8:2657. doi: 10.3389/fmicb.2017.02657 (PMC5767591; doi:10.3389/fmicb.2017.02657)
Supplement: Supplementary file 2 [file Table_2.PDF]

Table S2. Presence of putative LpxE, LptA, and LpxO among *Brucella* spp.

Code	Strain	*lptA*	*lpxE/pgpB*	*lpxO/olsC*
bme	*Brucella melitensis bv. 1 16M*	✓ *BmelptA*	✓[1]	✗[2]
bmi	*Brucella melitensis ATCC 23457*	✓	✓	na
bmz	*Brucella melitensis M28*	✓	✓	✗
bmg	*Brucella melitensis M5-90*	✓	✓	✗[3]
bmw	*Brucella melitensis NI*	✓	✓	✗[3]
bmee	*Brucella melitensis bv. 3 Ether*	✓	✓	✗[3]
bmf	*Brucella abortus 2308*	✗	✓ *BalpxE/pgpB*	na
bmb	*Brucella abortus bv. 1 9-941*	✗	✓	na
bmc	*Brucella abortus S19*	✗	✓	✗[3]
baa	*Brucella abortus A13334*	✗	✓	✗
babo	*Brucella abortus bv. 2 86/8/59*	✗	✓	✗[3]
babr	*Brucella abortus bv. 6 870*	✗	✓	✗[3]
babt	*Brucella abortus 63 75*	✗	✓	✗[3]
babb	*Brucella abortus BDW*	✗	✓	✗[3]
babu	*Brucella abortus bv. 9 C68*	✗	✓	✗[3]
babs	*Brucella abortus BER*	✗	✓	✗[3]
babc	*Brucella abortus NCTC 10505*	✗	✓	✗[3]
bms	*Brucella suis 1330*	✓	✓	na
bsf	*Brucella suis bv. 1*	✓	✓	na
bsui	*Brucella suis bv. 2 Bs143CITA*	✓	✓	✗
bsup	*Brucella suis bv. 2 PT09143*	✓	✓	✗
bsuv	*Brucella suis bv. 2 PT09172*	✓	✓	✗
bsuc	*Brucella suis bv. 2 Bs364CITA*	✓	✓	✗
bmt	*Brucella suis ATCC 23445*	✓	✓	na
bsz	*Brucella suis bv. 3*	✓	✓	✗[3]
bsv	*Brucella suis VBI22*	✓	✓	✗[3]
bsw	*Brucella suis ZW043*	✓	✓	✗[3]
bsg	*Brucella suis ZW046*	✓	✓	✗[3]
bov	*Brucella ovis*	✓	✓	na
bcs	*Brucella canis ATCC 23365*	✓	✓	na
bsk	*Brucella canis HSK A52141*	✓	✓	✗
bol	*Brucella canis Oliveri*	✓	✓	na
bcar	*Brucella canis RM6/66*	✓	✓	✗[3]
bcas	*Brucella canis SVA13*	✓	✓	✗[3]
bmr	*Brucella microti*	✓	✓	✓ *Bmilpx0/OlsC*
bpp	*Brucella pinnipedialis B2/94*	✓	✓	na
bpv	*Brucella pinnipedialis 6/566*	✓	✓	✗[3]
bcet	*Brucella ceti TE10759-12*	✓	✓	✗[3]
bcee	*Brucella ceti TE28753-12*	✓	✓	✗[3]
bvl	*Brucella vulpis*	✓	✓	✓[3]
oan	*Ochrobactrum anthropi ATCC 49188*	✓[4]	✓[5]	✓[3,6]

✓ Present

✗ Frameshift leading to a premature stop

1 Start annotated 60 pb downstream respect to *BalpxE/pgpB* start

2 Start annotated 189 pb downstream respect to *BmilpxO/olsC* start

3 Start annotated 105 pb downstream respect to *BmilpxO/olsC* start

4 Identity to *BmelptA* of 0.77

5 Identity to *BalpxE/pgpB* of 0.73

6 Identity to *BmilpxO/olsC* of 0.95
