# Supplementary material for: Identification of lptA, lpxE, and lpxO, Three Genes Involved in the Remodeling of Brucella Cell Envelope
Source: Front Microbiol. 2018 Jan 10;8:2657. doi: 10.3389/fmicb.2017.02657 (PMC5767591; doi:10.3389/fmicb.2017.02657)
Supplement: Supplementary file 3 [file Table_3.PDF]

Table S3. MALDI-TOF analysis of Bme-parental, *BmeΔlptA* and Ba-parental lipid A.

	Observed isotopic mass			Proposed molecule
	Bme-parental	*BmeΔlptA*	*Ba-parental*	
A	2173	2173	2173	$C_{120}H_{230}N_4O_{24}P_2$
A-Pi	2093	2093	2093	$C_{120}H_{229}N_4O_{21}P$
A+ (CH_2-CH_2)	2202	2002	2002	$C_{122}H_{234}N_4O_{24}P_2$
B	2073	2073	2073	?
B- (CH_2-CH_2)	2044	2044	2044	?
B- Pi	1993	1993	1993	?
B-Pi- (CH_2-CH_2)	1965	1965	1965	?
C	2145	2145	2145	$C_{118}H_{226}N_4O_{24}P_2$
C-Pi	2065	2065	2065	$C_{118}H_{225}N_4O_{21}P$
D	2191	2191	traces	$C_{120}H_{232}N_4O_{25}P_2$
D-Pi	2112	2112	No detected	$C_{120}H_{231}N_4O_{22}P$
D-Pi+PEA	2237	No detected	No detected	$C_{122}H_{238}N_5O_{25}P_2$
