# Supplementary material for: Identification of lptA, lpxE, and lpxO, Three Genes Involved in the Remodeling of Brucella Cell Envelope
Source: Front Microbiol. 2018 Jan 10;8:2657. doi: 10.3389/fmicb.2017.02657 (PMC5767591; doi:10.3389/fmicb.2017.02657)
Supplement: Supplementary file 4 [file Image_1.PDF]

```
BMI_I1970       MRFPRPTIASGPLCILTAIYLLAFTNASFWQKLGAYFHDHPQKLIVAGAAILLLHIALLL
BMEI0118        MRFPRPTIASGPLCILTAIYLLAFTNASFWQKLGAYFHDHPQKLIVAGAAILLLHIALLL
BAB             MRFPRPTIASGPLCILTAIYLLAFTNASFWQKLGAYFHDHPQKLIVAGAAILLLHIALLL
                *********** **:***:*******:****:* *** ******:*..*****:***:*:

BMI_I1970       VFSAKYVIKPVLIFMILVAAGGSYFTATFGTIITRSVVEATFTTTQAESNQLLTTGFILH
BMEI0118        VFSAKYVIKPVLIFMILVAAGGSYFTATFGTIITRSVVEATFTTTQAESNQLLTTGFILH
BAB             VFSAKYVIKPVLIFMILVAAGGSYFTATFGTIITRSVVEATFTTTQAESNQLLTTGFILH
                .************* * *****:*** ****** *.*****:***:***.:*:****:**

BMI_I1970       MVLYGLIPSLLIVWVRVKHRPLLRKIAVNTVAITACLMGAVVLIGSDFGSFSSMYREHRS
BMEI0118        MVLYGLIPSLLIVWVRVKHRPLLRKIAVNTVAITACLVGAVVLIGSDFGSFSSMYREHRS
BAB             MVLYGLIPSLLIVWVRVKHRPLLRKIAVNTVAITACLVGAVVLIGSDFGSFSSMYREHRS
                *:**.::*:*:*:**********:*: ****:* .**  *:.*****:************

BMI_I1970       DIMERLEPITPLKSTIQYAASNLKDNRVVMQPLGLDAKQTLAPLPSGKKLLTVIVVGETA
BMEI0118        DIMERLEPITPLKSTIQYAASNLKDNRVVMQPLGLDAKQTLAPLPSGKKLLTVIVVGETA
BAB             DIMERLEPITPLKSTIQYAASNLKDNRVVMQPLGLDAKQTLAPLPSGKKLLAVIVVGETA
                ****** * ******:** . :*:*.::***********:* *: ******:********

BMI_I1970       RAKNFSLYGYKQETNPELKKQDIVAFTNTVSCGTSTAVSVPCMFSPFTRAEYSSTKFRSS
BMEI0118        RAKNFSLYGYKQETNPELEKQDIVAFTNTVSCGTSTAVSVPCMFSPFTRAEYSSTKFRSS
BAB             RAKNFSLYGYKQETNPELKNR---------------------------------------
                **:******* :******:::

BMI_I1970       ENLMDVLNHAGVKVAWYENNTGSKNVADRIAMVDLQNSGDTRYCKGGECLDQILVDYLKE
BMEI0118        ENLMDVLNHAGVKVAWYENNTGSKNVADRIAMVDLQNSGDTRYCKGGECLDQILVDYLKE
BAB             ------------------------------------------------------------

BMI_I1970       HLKNFNGNATIVLHMTGSHGPAYYRRYPAEFARFQPECRTSEFSNCTNEKIVNAYDNTIL
BMEI0118        HLKNFNGNATIVLHMTGSHGPAYYRRYPAEFARFQPECRTSEFSNCTNEKIVNAYDNTIL
BAB             ------------------------------------------------------------

BMI_I1970       YTDHILSEVIDLLKSNESRFAPAMIYMSDHGESLGENGLYLHAAPYFIAPNEQTHIPFIA
BMEI0118        YTDHILSEVIDLLKSNESRFAPAMIYMSDHGESLGENGLYLHAAPYFIAPNEQTHIPFIA
BAB             ------------------------------------------------------------

BMI_I1970       WFSPDYASATGLDTACLKQGAASPASHDNLFHTVLGMMGVKTSVYDATLDRFASCRKAAV
BMEI0118        WFSPDYASATGLDTACLKQGAASPASHDNLFHTVLGMMGVKTSVYDATLDRFASCRKAAV
BAB             ------------------------------------------------------------

BMI_I1970       ASQS
BMEI0118        ASQS
BAB             ----
```

Figure S1: Aligment of predicted LptA sequences from *B. microti* (BMI_I1970), *B. melitensis* (BME BMEI0118) and *B. abortus* (BAB). Lpta from *B. microti* and *B.melitensis* conserve the predicted functional domains (Næssan et al, 2008) PD005703: GE[ST]x(9)Gx(26-37)T /// Dx(21-49)HxxGxH and PD461453: Yx(7)Dx(21-24)YxxDHGx(22-26)P shadowed in green and yellow, respectively, wheras Lpta from *B. abortus* lacks both domains.
