# Supplementary material for: Identification of lptA, lpxE, and lpxO, Three Genes Involved in the Remodeling of Brucella Cell Envelope
Source: Front Microbiol. 2018 Jan 10;8:2657. doi: 10.3389/fmicb.2017.02657 (PMC5767591; doi:10.3389/fmicb.2017.02657)
Supplement: Supplementary file 5 [file Image_2.pdf]

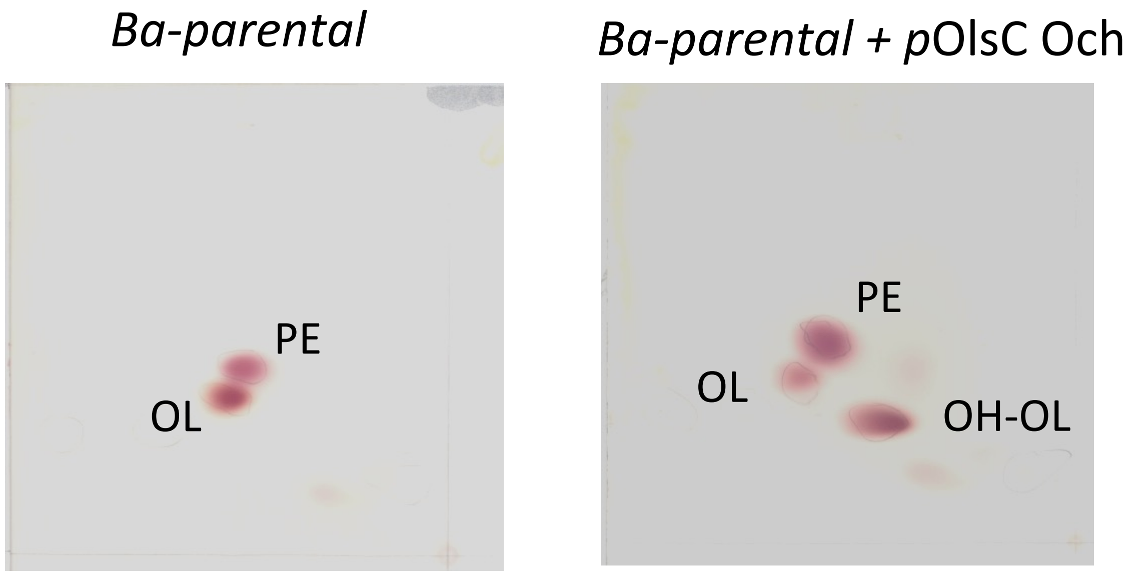

Figure S2. Introduction of *O.anthropi* OlsC into Ba-parental ind uces the hydroxylation of OL . Bidimensional chromatography of total lipid extracts followed by ninhydrin staining for aminolipids.
